# Endothelin-1–Mediated Alteration of Metallothionein and Trace Metals in the Liver and Kidneys of Chronically Diabetic Rats

**DOI:** 10.1080/15604280214281

**Published:** 2002

**Authors:** Lu Cai, Shali Chen, Terry Evans, M. George Cherian, Subrata Chakrabarti

**Affiliations:** 1 Department of Pathology The University of Western Ontario Dental Sciences Building London, Ontario N6A 5C1 Canada

## Abstract

In the present study, the role of endothelin-1 (ET-1) on alterations of hepatic and renal metallothionein (MT) and trace metals (Zn, Cu, and Fe) were investigated in streptozotocin (STZ)- induced diabetic rats. Diabetic rats, age- and sex-matched controls, as well as control and diabetic animals on a dual ET_A_/ET_B_ receptor blocker, bosentan, were investigated after 6 months of follow-up. MT was measured by cadmium-heme assay. Metals were measured by atomic absorption spectrometer. ET-1 mRNA was analyzed by reverse transcriptase–polymerase chain reaction (RT-PCR) technique. Hepatic and renal ET-1 mRNA was increased in diabetic rats as compared to control rats, along with an increase in both hepatic and renal MT proteins. The increased hepatic MT protein level was associated with decreases in hepatic Cu and Fe, whereas increased renal MT was associated with increases in renal Cu and Fe accumulation. Zn levels were unaltered in both organs in diabetic rats. Bosentan treatment partially prevented the increase in MT levels in both liver and kidney, along with reduced serum creatinine and increased urinary creatinine levels. Further bosentan treatment corrected the increased Cu and Fe levels in the kidney in diabetic rats, but reduced hepatic Cu and Fe levels. No significant effects of bosentan treatment on nondiabetic rats were observed. The data suggest that the possible effects of ET antagonism in diabetes may be mediated via changes in MT and trace metals.


*Int. Jnl. Experimental Diab. Res.*, 3:193–198, 2002
Copyright © 2002 Taylor & Francis
1560-4284/02 $12.00 + .00
DOI: 10.1080/15604280290013900

Taylor & Francis healthsciences

### Endothelin-1–Mediated Alteration of Metallothionein and Trace Metals in the Liver and Kidneys of Chronically Diabetic Rats

**Lu Cai, Shali Chen, Terry Evans, M. George Cherian, and Subrata Chakrabarti**

*Department of Pathology, The University of Western Ontario, London, Ontario, Canada*

**In the present study, the role of endothelin-1 (ET-1) on alterations of hepatic and renal metallothionein (MT) and trace metals (Zn, Cu, and Fe) were investigated in streptozotocin (STZ)-induced diabetic rats. Diabetic rats, age- and sex-matched controls, as well as control and diabetic animals on a dual $ET_A/ET_B$ receptor blocker, bosentan, were investigated after 6 months of follow-up. MT was measured by cadmium-heme assay. Metals were measured by atomic absorption spectrometer. ET-1 mRNA was analyzed by reverse transcriptase–polymerase chain reaction (RT-PCR) technique. Hepatic and renal ET-1 mRNA was increased in diabetic rats as compared to control rats, along with an increase in both hepatic and renal MT proteins. The increased hepatic MT protein level was associated with decreases in hepatic Cu and Fe, whereas increased renal MT was associated with increases in renal Cu and Fe accumulation. Zn levels were unaltered in both organs in diabetic rats. Bosentan treatment partially prevented the increase in MT levels in both liver and kidney, along with reduced serum creatinine and increased urinary creatinine levels. Further bosentan treatment corrected the increased Cu and Fe levels in the kidney in diabetic rats, but reduced hepatic Cu and Fe levels. No significant effects of bosentan treatment on nondiabetic rats were observed. The data suggest that the possible effects of ET antagonism in diabetes may be mediated via changes in MT and trace metals.**

**Keywords** Diabetes; Endothelin-1; Metallothionein; Trace Metals

In diabetic patients, the deranged regulation of hemodynamics may occur in a variety of organs [1, 2]. Vasoactive factors that are perturbed under hyperglycemic conditions may lead to altered tissue hemodynamics. A group of vasoactive proteins called endothelins (ETs) have been implicated in the pathogenesis of diabetes-induced vascular dysfunction in various organs [3–5]. ETs are a family of 21–amino acid peptides that include ET-1, ET-2, and ET-3, and interact in mammals with a group of receptors namely, $ET_A$ and $ET_B$ [6, 7]. ETs, originally discovered as endothelial products, have since been found in other organs, including liver and kidney [6–8]. ETs and their receptors were found to be altered in various target organs of diabetic complications in rats, suggesting the possible involvement of ETs in the pathogenesis of diabetic complications [3–5, 9, 10]. Along with their important roles in modulation of tissue blood flow, ET-1 is also able to stimulate cell proliferation by acting as a mitogenic factor [6, 7].

We have previously demonstrated that high glucose–induced augmentation of ET-1 expression in endothelial cells may regulate the expression of other stress-responsive proteins such as metallothionein (MT) [11]. MTs are a group of low-molecular-weight (6000 to 7000 Da), cysteine-rich (30%) intracellular proteins with high affinity for zinc (Zn) and copper (Cu). Four major isoforms of MT have been identified in mammalian tissues; MT-I and MT-II isoforms being the 2 major isoforms found in most tissues. Several physiologic roles have been proposed for MT, including detoxification of potentially toxic metals such as cadmium (Cd) and storage of essential metals such as zinc and copper [12, 13]. MT is induced readily in the liver by a variety of factors such as glucocorticoids, bacterial lipopolysaccharide (LPS), interferon, alkylating agents, and radiation [12–14]. MT, in addition, as one of the stress-responsive proteins, can be induced by a variety of cytokines, such as interleukin-1 (IL-1), interleukin-6 (IL-6), and tumour necrosis factor-$\alpha$ (TNF$\alpha$), during immunological or inflammatory reactions and may therefore

Received 4 March 2002; accepted 23 May 2002.
This work was supported by grants from the Canadian Diabetes Association in honor of the late Margaret Francis and from the Canadian Institute of Health Research (MOP43841).
Address correspondence to Dr. Subrata Chakrabarti, Department of Pathology, University of Western Ontario, Dental Sciences Building, London, Ontario, Canada N6A 5C1. E-mail: schakrab@uwo.ca be of importance in several disease processes [12–15]. MT levels are also increased in response to chronic stresses such as allogenic and isogenic organ transplantation [16, 17]. There are a few reports on increased levels of MT in kidney and liver of diabetic rats [18–20].

The aim of the present study was to investigate whether, similar to our previous findings in endothelial cells, chronic diabetes affects MT expression in the liver and kidney by an ET-dependent pathway, and if such an alteration may modulate trace metal accumulation in these organs.

### MATERIALS AND METHODS

### Animals and Treatments

All animals were cared for in accordance with the University of Western Ontario Council on Animal Care Committee, who formally approved all experimental protocols. Male Sprague-Dawley rats of approximately 200 g were obtained from Charles River Canada (St. Constant, PQ, Canada) and were given regular rat chow and water ad libitum. The rats were randomly divided into 4 groups: namely, nondiabetic control rats (C), diabetic rats (D), diabetic rats treated with bosentan (DB), and nondiabetic control rats treated with bosentan (CB). Bosentan, a potent orally active dual $ET_A/ET_B$ receptor blocker, was obtained from Actelion (Allschwill, Switzerland, courtesy of Dr. M. Clozel) [21].

Diabetes was induced with a single intravenous injection of streptozotocin (STZ, 65 mg/kg body weight in citrate buffer; Sigma, St. Louis, MO). The control animals received the same volume of buffer injection alone. Diabetic animals were monitored daily with respect to urine volume, sugar, and ketones, and received a daily dose of insulin (∼2 U/day) to prevent ketosis. Body weight and blood glucose levels were monitored regularly. Bosentan was administered by daily oral gavage at the dose of 100 mg/kg body weight/day. Animals from each group were sacrificed after 6 months of follow-up. Urine and serum creatinine levels were measured in the urine and blood samples collected at the time of sacrifice. The animals were anesthetized with sodium pentobarbital (50 mg/kg, intraperitoneal [IP]) and were sacrificed by cardiac puncture. Liver and kidney tissues were removed. Part of the right lobe of the liver and right renal cortex from each animal were snap frozen in liquid nitrogen and were stored at −80°C until analysis of mRNA. The remaining portion of the right lobe of liver and right renal cortex were frozen and used for analysis of MT protein and metal levels.

### MT Measurement by Cadmium-Hemoglobin Assay

MT levels in hepatic and renal tissues were determined by a cadmium-hemoglobin (Cd-heme) assay as previously described by us [22]. Briefly, the dissected tissues were homogenized in 0.25 M sucrose and centrifuged at 20,000 rpm for 20 minutes. An aliquot of the resulting supernatant fraction, diluted with 30 mM Tris-HCl buffer (pH 8.0), was incubated with 10 ppm $^{109}$Cd solution with known specific activity to saturate the metal-binding sites of MT. Excess Cd was removed by addition of rat hemolysate to the assay tubes followed by heat treatment in a boiling water bath, which caused precipitation of Cd-heme and other proteins, except MT (which is heat stable). The denatured proteins were removed by centrifugation at 10,000 rpm for 2 minutes. Hemolysate treatment/heat denaturation/centrifugation steps were repeated 3 times. The Cd concentrations in the final supernatant were calculated from the radioactivity of the $^{109}$Cd, which was measured by a $\gamma$ counter (1272 Clinigamma, LKB Wallac; Turku, Finland), and was converted to MT concentration on the basis of 7 g-atoms of cadmium/MT. The total hepatic and renal MT concentrations were expressed as micrograms per gram of wet tissue.

### Metal Quantification

Zn, Cu, and Fe concentrations of liver and kidneys were determined by an atomic absorption spectrometry (Varian Spectra-AA 30, Georgetown, Ontario, Canada) using an air-acetylene flame after tissue digestion, as previously described by us [22]. Preweighed pieces of tissue samples were digested in nitric acid at room temperature overnight and then heated to 95°C for 1 hour to facilitate digestion. The resulting clear liquid was used for analysis. Zn, Cu, and Fe concentrations were expressed as micrograms per gram wet tissue.

### ET-1 mRNA Measurement

TRIZOL reagent (Canadian Life Technologies, Burlington, Ontario, Canada) was used to isolate RNA from the tissues as described by manufacturer's instruction. Quantitation of RNA was performed by determining the absorbance at 260 nm and 280 nm. First strand cDNA synthesis was performed using Superscript-II system (Canadian Life Technologies) as previously described [3]. The amplification was carried out using our previously described methodologies [3]. The ET-1 primers, 5′-GCT CCT GCT CCT CCT TGA TG-3′ (sense) and 5′-CTC GCT CTA TGT AAG TCA TGG-3′ (antisense), with a predicted product size of 499 bp, were used. Reactions were performed in 30-$\mu$l volumes containing 1× polymerase chain reaction (PCR) buffer, 1.5 mM $MgCl_2$, 250 $\mu$M dNTP mix, 1 $\mu$M of each amplification primer, 2.5 U *Taq* polymerase, and 4 $\mu$l of the reverse transcriptase (RT) product. The initial cycle was carried out using 3 minute at 94°C for denaturation, 1 minute at 60°C for annealing, and 3 minutes at 72°C for extension. Subsequent cycles of PCR were performed using the following conditions: denaturation, 45 seconds at 94°C; annealing, 45 seconds at 54°C; and extension, 1 minute at 72°C; and 7 minutes for final extension. The linearity of the PCR reaction was established by analyzing the PCR product with variable amount of template and variable cycle numbers. It has been previously shown that in this reaction the PCR amplification is log-linear up to 40 cycles. In this study, we used 30 cycles of amplification. Simultaneously a housekeeping gene ($\beta$-actin) was amplified in a separate set of tubes using the same RT product. The primer sequences for $\beta$-actin are 5′-TGG TGG TAT GGG TCA GAA GG-3′ (sense) and 5′-ATC CTG TCA GCG ATG CCT GGG-3′ (antisense), with a predicted product size of 813 bp. The amplification products were analyzed on a 2.5% agarose gel, stained with ethidium bromide, and visualized under ultraviolet (UV) light. Quantitation was performed by serial dilution slot-blot hybridization and densitometry of the products from the upstream of amplification onto the nylon membranes. Hybridizations were performed with biotinylated amplification product specific oligoprobes (ET-1: 5′-CAA AGA CCA CAG ACC AAG GG-3′ and $\beta$-actin: 5′-CTG ACC CTG AAG TAC CCC ATT-3′) [3]. The detection was carried out using a NBT/BCIP system (Sure Blot, Oncor, Gathisburg, MD, USA). The blots were analyzed by a Hewlett-Packard 4C scanner and Mocha Image Analysis Software (Jandel Scientific, San Rapael, CA, USA). Each sample was analyzed in duplicate. The densitometric values were expressed in arbitrary units per microgram of total RNA and the ratio of $\beta$-actin to ET-1 was obtained as previously described [3].

### Statistical Analysis

Data are expressed as mean ± SE, with a minimum of 5 rats per group. The data were subject to unpaired 2-tail Student's *t* test. A *P* value of 0.05 or less was accepted as being significant.

### RESULTS

### Clinical Parameters

Diabetic rats showed glucosuria (>2%), hyperglycemia, and reduced body weight, compared to the age- and sex-matched control animals (data not shown). Bosentan treatment had no effect on these parameters. Diabetic animals showed increased serum creatinine levels (D: 5.6 ± 0.8 mg/dl versus C: 4.0 ± 0.5 mg/dl, $P < 0.05$), which were corrected by bosentan treatment (DB: 3.3 ± 0.6 mg/dl, $P < 0.05$).

### MT Protein and Metal Alterations in Diabetic Rats

Hepatic as well as renal MT proteins were significantly increased in STZ-diabetic rats at 6 months after diabetes (Figure 1).

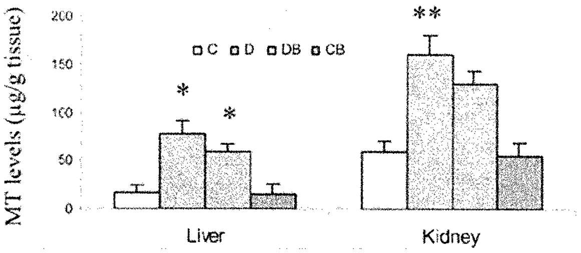

**FIGURE 1**
MT protein levels in livers and kidneys of control (C), diabetic (D), bosentan-treated diabetic (DB), and bosentan-treated control rats (CB) after 6 months of follow-up. Data are presented as mean ± SE, n = 6 in all groups. *Significantly different from C; **significantly different from C and DB.

The hepatic Zn, Cu, and Fe levels were significantly ($P < 0.05$) decreased in the diabetic rats at 6 months (Figure 2). In contrast, renal Cu and Fe levels were increased ($P < 0.05$) in diabetic rats, with no change in renal Zn levels (Figure 2).

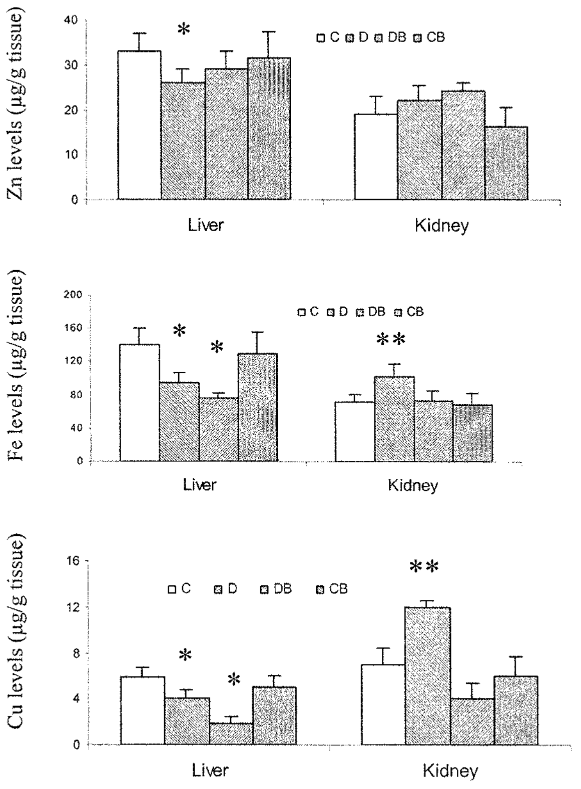

**FIGURE 2**
Zn, Fe, and Cu levels in livers and kidneys of control (C), diabetic (D), bosentan-treated diabetic (DB), and bosentan-treated control rats (CB) after 6 months of follow-up. Data are presented as mean ± SE, n = 6 in all groups. *Significantly different from C; **significantly different from C and DB.

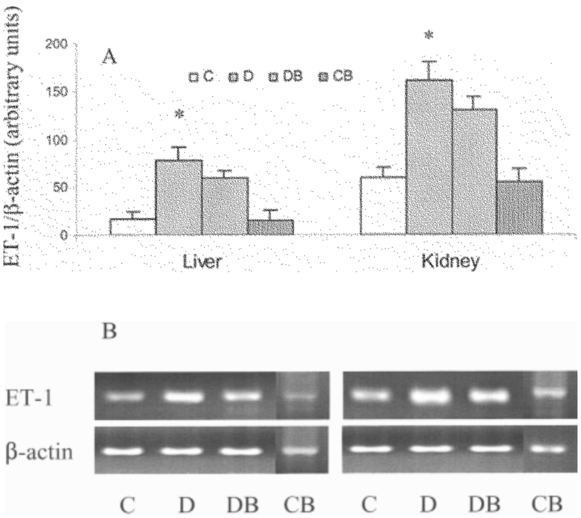

**FIGURE 3**
mRNA analysis of ET-1 by semiquantitative RT-PCR from livers and kidneys of control (C), diabetic (D), bosentan-treated diabetic (DB), and bosentan-treated control rats (CB) after 6 months of follow-up. Data are expressed as the ratio of ET-1 gene to housekeeping gene, $\beta$-actin. Lower panel shows representative gel pictures. *Significantly different from C.

### ET-1 mRNA Expression in Diabetic Rats

Compared to the nondiabetic control animals, diabetic rats showed increased expression of ET-1 mRNA in the liver and kidney (Figure 3). As there are no intracellular storage mechanism for ET-1, the changes in mRNA levels are reflective of protein levels [6, 7]. Bosentan treatment had no effect on ET-1 mRNA expression in diabetic or control rats.

### Effects of Bosentan on MT and Metal Alterations

In the liver, increased MT protein in diabetic rats was not prevented by the administration of bosentan (Figure 1). ET receptor blockade with bosentan tended to restore the reduced Zn levels in the liver of diabetic rats. However, with respect to hepatic Cu and Fe, it further aggravated the diabetes-induced reduction of these 2 metals (Figure 2). In contrast, in the kidneys, although bosentan tended to decrease MT protein levels (Figure 1), it completely restored the diabetes-induced increases in Cu and Fe levels. No effect of bosentan on renal Zn levels were seen (Figure 2). No effects of bosentan treatment on MT or metal levels were noted in control animals in any of the organs studied.

### DISCUSSION

Hyperglycemia may trigger several alterations in metabolic events, including the abnormal metabolism of trace elements and related proteins in various organs [18–20]. In this study, we have demonstrated that alterations in metal-binding proteins, in both the kidney and liver, at least in part, may be regulated via ETs. However, the pattern of associated effects on metal accumulation varied in these 2 organs; in contrast to reduced Cu and Fe levels in the liver, the kidney showed increased levels. Some of these changes were corrected by ET antagonism with bosentan.

These findings demonstrate a novel mechanism of regulation of metal-binding proteins and metal distribution in diabetic animals. These data are in keeping with our previous findings in endothelial cells, which showed that high glucose increases both MT mRNA and protein expression and that these changes are regulated by an ET-dependent pathway [11]. Although other investigators have demonstrated that ET-1 induces MT expression in endothelial cells under normal glucose culture conditions [23], in this study, no effects of ET antagonism on MT levels or metal accumulation were seen in control rats.

Although the liver showed increased MT expression in diabetic rats, this was associated with decreased total hepatic Zn, Cu, and Fe levels. These findings are in keeping with previous studies and is probably related to decreased absorption of metals such as Zn, Cu, and Fe from intestinal mucosa in diabetic rats [19]. However, the increase in MT suggests that the most of Zn and Cu may be bound to MT.

In contrast, increased renal MT was accompanied by increased accumulation of renal Cu and Fe. Similar results have been reported in STZ-diabetic rats [19]. Bosentan completely prevented the increase in renal Cu and Fe in diabetic rats, and reduced renal MT protein. Because Zn levels were not altered, these changes may not be related to food intake.

Although the biological functions of MTs remain elusive, there are several lines of evidence to suggest important roles for this protein in various disease conditions, and could be related to its effects on homeostasis of metals, apoptosis, and modulation of nitric oxide (NO) activity [24, 25]. MT may regulate NO signaling in endothelial cells [26, 27]. On the other hand, there is a feedback regulatory mechanism of NO or ET [28, 29]. Hence, one may speculate that a regulatory mechanism exists, with interactions among NO, ET, and MT, which targets vascular functions in diabetes. However, further confirmation is needed.

Increased renal Cu levels in chronic diabetes, as shown in this study, is in keeping with previous data [19, 20]. The exact significance of this finding is unclear. It is not known whether increased glomerular filtration in diabetes may in any way contribute to trace metal alteration. It has, however, been postulated that increased Cu in the kidney may effect the activity of the copper lysyl oxidase enzyme, which is a significant component in the regulation of basement membrane turnover [30]. It is of interest to note that recent data demonstrate that there is a duration-dependent alteration in the Cu/Zn ratio in STZ-diabetic rats, and that increased Cu/Zn ratio in diabetic patients is associated with an increased incidence of vascular complications

[31, 32]. Increased Cu has been associated with cardiovascular disease in patients with end-stage renal failure [33]. Furthermore, increased Cu has been shown to augment the formation of advanced glycosylation end products and act as an inhibitor to aminoguanidine [34]. We and others have previously demonstrated that ETs are of importance in the pathogenesis of diabetic nephropathy by modulating growth factors and extracellular matrix protein production [10, 35]. The effect of ET antagonism on the serum creatinine levels in the diabetic rats is in keeping with these observations. The present data suggest that one mechanism of ET blockade in the kidney may be mediated by changes in trace metals.

### REFERENCES

[1] King, G. L., and Brownlee, M. (1996) The cellular and molecular mechanisms of diabetic complications. *Endocrinol. Metab. Clin. North. Am.,* **25,** 255–270.

[2] Williamson, J. R., Chang, K., Frangos, M., et al. (1993) Perspectives in diabetes hyperglycemic pseudohypoxia and diabetic complications. *Diabetes,* **42,** 801–813.

[3] Deng, D. X., Evans, T., Mukherjee, K., Downey, D., and Chakrabarti, S. (1999) Diabetes induced vascular dysfunction in the retina: Role of endothelins. *Diabetologia,* **42,** 1228–1234.

[4] Sarman, B., Toth, M., and Somogyi, A. (1998) Role of endothelin-1 in diabetes mellitus. *Diabetes Metab. Rev.,* **14,** 171–175.

[5] Chakrabarti, S., Cukiernik, M., Hileeto, D., Evans, T., and Chen, S. (2000) Role of vasoactive factors in the pathogenesis of early changes in diabetic retinopathy. *Diabetes Metab. Res. Rev.,* **16,** 393–407.

[6] Rubanyi, G. M., and Polokoff, M. A. (1994) Endothelins: Molecular biology, biochemistry, pharmacology, physiology and pathophysiology. *Pharmacol. Rev.,* **46,** 325–414.

[7] Levin, E. R. (1995) Endothelins. *N. Engl. J. Med.,* **333,** 356–363.

[8] Yanagisawa, M., Kurihara, H., and Kimura, S. (1988) A novel potent vasoconstrictor peptide produced by vascular endothelial cells. *Nature,* **332,** 411–415.

[9] Chen, S., Evans, T., Mukherjee, K., Karmazyn, M., and Chakrabarti, S. (2000) Diabetes-induced myocardial structural changes: Role of endothelin-1 and its receptors. *J. Mol. Cell Cardiol.,* **32,** 1621–1629.

[10] Chen, S., Evans, T., Deng, D., Cukiernik, M., and Chakrabarti, S. (2001) Hyperhexosemia induced functional and structural changes in the kidney: Role of endothelins. *Nephron,* **90,** 86–94.

[11] Apostolova, M. D., Chen, S., Chakrabarti, S., and Cherian, M. G. (2001) High-glucose-induced metallothionein expression in endothelial cells: An endothelin-mediated mechanism. *Am. J. Physiol. Cell Physiol.,* **281,** C899–C907.

[12] Cherian, M. G. (1993) Biological functions of metallothionein—a review. In *Metallothionein III, Biological Roles and Medical Implications,* edited by K. T. Suzuki, N. Imura, and M. Kimura, pp. 87–104. Basel: Birkhauser Verlag.

[13] Cai, L., Satoh, M., Tohyama, C., and Cherian, M. G. (1999) Metallothionein in radiation exposure: Its induction and protective role. *Toxicology,* **132,** 85–98.

[14] Sato, M., and Bremner, I. (1993) Oxygen free radicals and metallothionein. *Free Radic. Biol. Med.,* **14,** 325–337.

[15] Naganuma, A., Satoh, M., and Imura, N. (1993) Utilization of metallothonein inducer in cancer therapy. In *Metallothionein III, Biological Roles and Medical Implications,* edited by K. T. Suzuki, N. Imura, and M. Kimura, pp. 255–268. Basel: Birkhauser Verlag.

[16] Courtade, M., Carrera, G., Paternain, J. L., Martel, S., Carre, P. C., Folch, J., and Pipy, B. (1998) Metallothionein expression in human lung and its varying levels after lung transplantation. Toulouse Lung Transplantation Group. *Chest,* **113,** 371–378.

[17] Cai, L., Deng, D. X., Jiang, J., Chen, S., Zhong, R., Cherian, M. G., and Chakrabarti, S. (2000) Induction of metallothionein synthesis with preservation of testicular function in rats following long term renal transplantation. *Urol. Res.,* **B28B,** 97–103.

[18] Chen, M. L., and Failla, M. L. (1988) Metallothionein metabolism in the liver and kidney of the streptozotocin-diabetic rat. *Comparat. Biochem. Physiol. B: Comparat. Biochem.,* **90,** 439–445.

[19] Craft, N. E., and Failla, M. L. (1983) Zinc, iron, and copper absorption in the streptozotocin-diabetic rat. *Am. J. Physiol.,* **244,** E122–E128.

[20] Failla, M. L., and Kiser, R. A. (1983) Hepatic and renal metabolism of copper and zinc in the diabetic rat. *Am. J. Physiol.,* **244,** E115–E121.

[21] Roux, S., Breu, V., Ertel, S. L., and Clozel, M. (1999) Endothelin antagonism with bosentan: A review of potential applications. *J. Mol. Med.,* **77,** 364–376.

[22] Onosaka, S., and Cherian, M. G. (1981) The induced synthesis of metallothionein in various tissues of rat in response to metals. I. Effect of repeated injection of cadmium salts. *Toxicology,* **22,** 91–101.

[23] Kaji, T., Yamamoto, C., Tsubaki, S., Ohkawara, S., Sakamoto, M., Sato, M., and Kozuka, H. (1993) Metallothionein induction by cadmium, cytokines, thrombin and endothelin-1 in cultured vascular endothelial cells. *Life Sci.,* **53,** 1185–1191.

[24] Waalkes, M. P. (1993) Medical implications of metallothionein. In *Metallothionein III, Biological Roles and Medical Implications,* edited by K. T. Suzuki, N. Imura, and M. Kimura, pp. 243–254. Basel: Birkhauser Verlag.

[25] Cherian, M. G. (1994) The significance of the nuclear and cytoplasmic localization of metallothionein in human liver and tumor cells. *Environ. Health Perspect.,* **102** (Suppl 3), 131–135.

[26] Aravindakumar, C. T., Ceulemans, J., and De Ley, M. (1999) Nitric oxide induces $Zn^{2+}$ release from metallothionein by destroying zinc-sulphur clusters without concomitant formation of S-nitro-sothiol. *Biochem. J.,* **344,** 253–258.

[27] Pearce, L. L., Gandley, R. E., Han, W., Wasserloos, K., Stitt, M., Kanai, A. J., McLaughlin, M. K., Pitt, B. R., and Levitan, E. S. (2000) Role of metallothionein in nitric oxide signaling as revealed by a green fluorescent fusion protein. *Proc. Natl. Acad. Sci. U.S.A.,* **97,** 477–482.

[28] Vanhoutte, P. M. (1994) Endothelin-1. A matter of life and breath. *Nature,* **368,** 693–694.

[29] Chen, S., Apostolova, M. D., Cherina, M. G., and Chakrabarti, S. (2000) Interaction of endothelin-1 with vasoactive factors in mediating glucose induced increased permeability in the endothelial cells. *Lab. Invest.,* **80,** 1311–1321.

[30] Siegel, R. C., Pinell, S. R., and Martin, G. R. (1970) Cross linking of collagen and elastin. Properties of laysyl oxidase. *Biochemistry,* **9,** 4486–4492.

[31] Aguilar, M. V., Laborda, J. M., Martinex-Para, M. C., Gonzalez, M. J., Meseguer, I., Bernao, A., and Mateos, C. J. (1998) Effect of diabetes on the tissular Zn/Cu ratio. *J. Trace Elem. Med. Biol.*, **12,** 155–158.
[32] Karahan, S. C., Deger, O., Orem, A., Ucar, F., Erem, C., Alver, A., and Onder, E. (2001) The effects of impaired trace element status on polymorphonuclear leukocyte activation in the development of vascular complications in type 2 diabetes mellitus. *Clin. Chem. Lab. Med.*, **39,** 109–115.
[33] Healy, H., Reith, D., Morgan, C., Clague, A., and Westhuyzen, J. (2000) Are metalloproteins and acute phase reactants associated with cardiovascular disease in end-stage renal failure? *Ann. Clin. Lab. Sci.*, **30,** 295–304.
[34] Jakus, V., Bauerova, K., and Rietbrock, N. (2001) Effect of aminoguanidine and copper (II) ions on the formation of advanced glycosylation end products. In vitro study on human serum albumin. *Arzneimitteforschung*, **54,** 280–283.
[35] Nakamuta, T., Ebihara, I., Fukui, M., Tomino, Y., and Koide, H. (1995) Effect of a specific endothelin A receptor antagonist on mRNA levels for extracellular matrix components and growth factors in diabetic glomeruli. *Diabetes,* **44,** 895–899.

